# Association of Exposure to Intimate Partner Violence With Maternal Depressive Symptoms and Early Childhood Socioemotional Development Among Mothers and Children in Rural Tanzania

**DOI:** 10.1001/jamanetworkopen.2022.48836

**Published:** 2022-12-29

**Authors:** Clariana Vitória Ramos de Oliveira, Christopher Robert Sudfeld, Alfa Muhihi, Dana Charles McCoy, Wafaie W. Fawzi, Honorati Masanja, Aisha K. Yousafzai

**Affiliations:** 1 School of Nursing, University of Nevada, Las Vegas; 2 Department of Global Health and Population, Harvard T. H. Chan School of Public Health, Boston, Massachusetts; 3 Harvard Graduate School of Education, Cambridge, Massachusetts; 4 Ifakara Health Institute, Dar es Salaam, Tanzania

## Abstract

**Importance:**

Approximately 1 in 4 women experience intimate partner violence (IPV) or nonpartner sexual violence during their lifetime. Mothers exposed to IPV are more likely to experience depressive symptoms and to discipline their children harshly, which may affect their children’s socioemotional development; however, there is limited evidence on these outcomes.

**Objective:**

To examine the association between IPV, maternal depressive symptoms, harsh child discipline, and child stimulation with child socioemotional development.

**Design, Setting, and Participants:**

This study used cross-sectional follow-up data collected from February 19 to October 10, 2014, from a birth cohort of children aged 18 to 36 months who were enrolled in a randomized, double-blind, placebo-controlled trial of neonatal vitamin A supplementation in the Morogoro region of Tanzania. Data analysis occurred between September 10, 2019, and January 20, 2020.

**Exposures:**

Lifetime experience of IPV was assessed using an abbreviated module of the Tanzania Demographic and Health Survey, maternal depressive symptoms were assessed with the Patient Health Questionnaire, and data on harsh child discipline and maternal stimulation of their children were collected using modules of the United Nations Children's Fund Multiple Indicator Cluster Survey.

**Main Outcomes and Measures:**

Child socioemotional development was measured by the Caregiver-Reported Early Childhood Development Instruments.

**Results:**

A total of 981 mother-child dyads were included in the analytic sample; 388 children (39.6%) were between ages 18 and 24 (mean [SD] age, 27.06 [6.08]) months, and 515 (52.5%) were male children. A negative association was observed between maternal report of physical IPV only (mean difference, −0.022; 95% CI, −0.045 to −0.006) and physical and sexual IPV (mean difference, −0.045; 95% CI, −0.077 to −0.013) with child socioemotional scores, but neither was statistically significant after including depressive symptoms in the model, which is consistent with mediation. Furthermore, a negative association was observed between maternal mild to severe depressive symptoms and child socioemotional development, including adjustment for IPV (mean difference, −0.073; 95% CI, −0.103 to −0.043). Harsh disciplinary practices and stimulation were not associated with child socioemotional development after adjusting for IPV, maternal depressive symptoms, and other factors.

**Conclusions and Relevance:**

The findings of this study suggest that maternal depressive symptoms may explain the negative association between IPV and child socioemotional development.

## Key Points

**Question:**

Is there an association between intimate partner violence, maternal depressive symptoms, harsh child discipline, child stimulation, and children’s socioemotional development in rural Tanzania?

**Findings:**

This cross-sectional study used data from 981 mothers and their children aged 18 to 36 months and noted that physical only and physical and sexual intimate partner violence were associated with lower child socioemotional development scores. An association was also observed between mild to severe maternal depressive symptoms and lower child socioemotional development scores.

**Meaning:**

The findings of this cross-sectional study suggest that children whose mothers experienced intimate partner violence or depressive symptoms appear to have poorer socioemotional development.

## Teaser

This cross-sectional study examines factors associated with the socioemotional development of children whose mothers experience intimate partner violence.

## Introduction

Global estimates indicate that approximately 1 in 4 women have experienced physical and/or sexual intimate partner violence (IPV) or nonpartner sexual violence during their lifetime.^1^ The prevalence of IPV in low- and middle-income countries is high, with a prevalence of 42% in Western Africa and South Asia and 66% in central sub-Saharan Africa.^2^ Women who experience IPV report higher rates of several substantial physical and mental health problems, including depression and posttraumatic stress disorder.^3^ In addition to affecting a woman’s health, IPV may present risks to the safety and well-being of children living in the household, including a higher risk of mortality^4^ and severe acute malnutrition.^5^ Furthermore, IPV has been shown to be associated with poorer early child development (ECD) outcomes.^6,7^ However, the association between sexual and physical IPV with socioemotional development is less clear.

In addition to the direct effects on stress and well-being that IPV may have on children who witness their mothers being abused,^8^ the association between IPV and ECD may be partially mediated by household and maternal factors, including an increased risk of maternal depression and suboptimal parenting practices. Intimate partner violence may serve as a source of trauma and stress for mothers, compromising their own well-being as well as their ability to respond sensitively and responsively to their children’s needs.^9^ For example, a study conducted in Tanzania observed that 30.0% of mothers who reported experiencing at least 1 type of IPV during their pregnancy had an increased risk of postpartum depression.^10^ Childcare practices can also be affected by IPV experience as IPV has been identified as a risk factor for harsh child discipline.^11^ In Tanzania specifically, a national survey observed that almost three-quarters of children had experienced physical disciplinary practices (eg, being kicked or spanked) and more than one-quarter had experienced emotional violence (eg, being yelled at and humiliated).^12^ In addition, a recent study reported that the negative association between IPV and overall ECD was mediated by lower levels of maternal and paternal stimulation (the engagement of caregivers in play and learning activities with their young children).^6^

Independent of IPV, studies have reported that maternal depressive symptoms are associated with lower levels of child socioemotional development^13^ and increased emotional regulation problems.^14^ Exposure to maternal depressive symptoms concurrent with IPV has also been noted to be associated with poor language and gross motor development.^15^ However, despite the volume of literature linking IPV to suboptimal development, there is comparatively limited evidence on the association between IPV, maternal depressive symptoms, and parenting practices with children’s socioemotional development.

**Figure 1.**
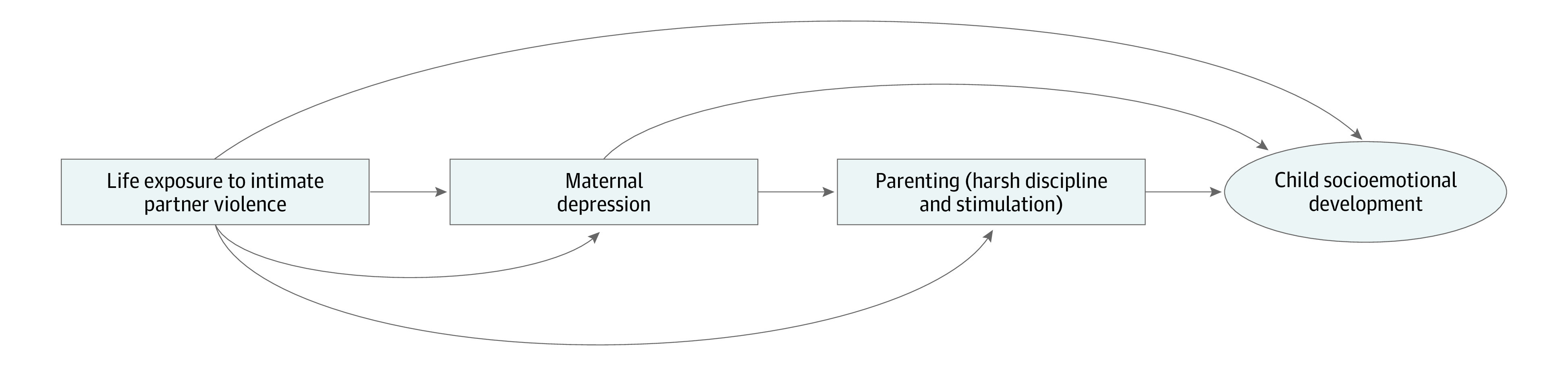
Conceptual Model

Given these evidence gaps, this study examined the association between IPV, maternal depressive symptoms, harsh child discipline, stimulation, and children’s socioemotional development in rural Tanzania. The hypothesis was that children’s early socioemotional development is associated with maternal experience of IPV, maternal depressive symptoms, and parenting practices (harsh disciplinary practices and early stimulation). The conceptual model we examined is shown in Figure 1.

## Methods

### Study Design

This analysis used data from a cross-sectional study of child development conducted among a birth cohort of children who were enrolled in a randomized, double-blind, placebo-controlled trial of neonatal vitamin A supplementation (NVAS), conducted in the Morogoro region of Tanzania.^16,17^ This secondary analysis report is aligned with the Strengthening the Reporting of Observational Studies in Epidemiology (STROBE) reporting guideline. The parent NVAS trial recruited and followed up infants to age 1 year from August 26, 2010, to March 3, 2014. A child development follow-up study was subsequently conducted when the children were aged 18 to 36 months from February 19 to October 10, 2014. The data analysis for the present study occurred between September 10, 2019, and January 20, 2020. Written informed consent was obtained from all participants in this study. All study protocols were approved by institutional review boards at the Harvard T. H. Chan School of Public Health, the National Institute of Medical Research of Tanzania, and the Ifakara Health Institute. Participants were reimbursed for transport expenses based on distance from their home to the study clinic.

### Study Participants

For the NVAS trial, a total of 31 999 randomly selected children living in Morogoro region were enrolled and followed up until age 1 year. For the child development follow-up study, sampling was restricted to a subset of children from the original trial living within the Ifakara Demographic Surveillance Site. The full cohort was not followed up due to logistic and financial reasons. Children were selected for participation in the child development study based on the following inclusion criteria: (1) age 18 to 36 months at the time of assessment, (2) lived in Ifakara town or surrounding villages, and (3) caregiver consented to participate.^16^ No data on race and ethnicity were recorded. The mother-child pairs included in the sample for this study were selected by a computer-generated random number drawn from the primary NVAS trial sample and received both a home visit and an invitation to an additional clinic visit, which occurred 1 to 6 days after the home visit for data collection activities. The analytic sample comprised 981 mothers who completed the IPV questionnaire, maternal depressive symptoms assessment, and childcare questionnaire.

### Study Data Collection

Trained field staff visited the home to administer a questionnaire to collect mothers’ demographic, socioeconomic, and environmental information, as well as their reports on parenting practices (disciplinary practices and stimulation) and child development using the Caregiver-Reported Early Childhood Development Instruments (CREDI),^16,18,19^ as explained with more details in the Measures section. Data collectors were 8 male, secondary school–educated field workers with previous experience in research with families and children in the area. The coordinators were provided with a 2-day training session on the CREDI and other study assessments. They supported the data collectors in the field once every 2 weeks to ensure continued adherence to study protocols. The data collectors observed the caregivers’ understanding of the CREDI items and recorded any questions or concerns stated by the caregivers during the interview.

### Measures

#### Children’s Socioemotional Development

Children’s socioemotional development was the primary outcome of interest and was measured using the CREDI.^16^ The CREDI is a caregiver-reported, low-cost, and culturally neutral measure of ECD in children younger than 3 years. The CREDI was validated for the Tanzania population with good acceptability and adequate reliability. Cronbach α coefficients calculated suggested acceptable internal consistency and interitem reliability, with an internal consistency for the socioemotional subscale of α = 0.68.^16^ The initial validation effort in Tanzania suggested that the CREDI tool may provide a valid method for capturing young children’s development across socioemotional domains and an acceptable tool for use in low-resourced settings.^16^ The socioemotional scale comprises 20 items on emotional and behavioral self-regulation, emotional knowledge, and social competence. For example, the field team asked the mother 2 questions: “Does the child involve others in play (ie, play interactive games with other children)?” “Does the child smile when others smile at him/her?” The CREDI is scored continuously using age-standardized scoring procedures that are based on children’s raw percentage yes (pass) responses within each age group. The CREDI average scores range from 0 to 1, with lower scores indicating lower development.

#### Intimate Partner Violence

Lifetime exposure to IPV was assessed using an abbreviated IPV module of the Tanzania Demographic and Health Survey.^20^ Four categories were considered: none when the mother did not experience any type of IPV, IPV physical (only) if the mother answered yes when asked about whether her partner had ever physically hurt her, IPV sexual (only) if the mother answered yes when asked whether her partner had ever forced her to have sexual intercourse or perform other sexual acts with him, and IPV physical and sexual when the mother answered yes for both questions.

#### Maternal Depressive Symptoms

The Patient Health Questionnaire-9 (PHQ-9) is a scale of maternal depressive symptoms in diverse contexts that scores 9 symptoms of depression.^21^ The scale has an estimated 90% to 94% sensitivity and a specificity of 75% to 99% for major depressive disorder (assessed with the Mini International Neuropsychiatric Interview) in various groups.^22,23^ The PHQ-9 has been used as a reliable assessment in primary care settings in sub-Saharan Africa.^7,24^ The PHQ-9 scores for depressive symptoms are calculated by taking a sum of all 9 items, which are rated on a 0 to 3 response scale (not at all = 0, several days = 1, more than half the days = 2, and nearly every day = 3). Maternal depressive symptoms were then categorized into the following groups: none to minimal (0-4), mild (5-9), moderate (10-14), moderate to severe (15-19), and severe (20-27).^21^ We merged into 2 categories none to minimal (0-4) and moderate to severe (10-27) due to the low number of participants who scored moderate to severe depressive symptoms (10 participants scored >9 points).

#### Harsh Child Discipline

Questions on disciplinary practices were taken from the early childhood development module of the United Nations Children's Fund Multiple Indicator Cluster Surveys (MICS), which have been used widely in Tanzania. The questions used in the MICS were drawn from a modified version of the Parent-Child Conflict Tactics Scale^25^ and asked the mother, “In the past 3 days, has an adult done any of the following with the child: yelled at or scolded him/her? Spanked or hit him/her?” These items were scored considering 0 when the mother’s answer was no and 1 when the answer was yes.

#### Maternal Stimulation

Maternal stimulation was defined by the mother’s report of whether the child’s mother engaged in 6 play and learning activities with the child in the past 3 days: reading books or looking at pictures; telling stories; taking the child outside of the home compound; playing with the child; naming, counting, or drawing things with the child; and talking with the child while working or doing homework. These series of questions, also drawn from the MICS, are widely used, reliable, and valid measures of parental stimulation with predictive validity for ECD outcomes.^26,27,28^ We created a total stimulation score by summing the number of activities that the mother did with the child (range, 0-6), with higher values indicating greater maternal stimulation and lower values indicating low stimulation. After that, we created categories for low (0-2 items), moderate (3-4 items), and high (5-6 items) stimulation.^29^

### Statistical Analysis

The cross-sectional associations between experience of IPV, maternal depression, harsh discipline, child stimulation, and early childhood CREDI socioemotional scores were examined. Normality of the socioemotional development variable was assessed with the Shapiro-Wilk test and indicated that the outcome of interest was normally distributed (*P* > .05).

We constructed univariable linear regression models to assess these associations. We then constructed 3 multivariable linear regression models based on our conceptualization of the associations between the exposures of interest and the children’s socioemotional development (Figure 1). All multivariable models also included adjustment for maternal age (15-24, 25-34, and 35-44 years), maternal educational attainment (primary, secondary, and college), maternal occupation (income-generating activity and no income-generating activity), paternal presence in the home, household wealth quintile, child age and child sex, and the randomized vitamin A or placebo regimen in the parent NVAS trial.

In the first multivariable model, which was the primary analysis, we estimated the association between lifetime experience of IPV as the variable and early child socioemotional development as the outcome. We then constructed secondary models to examine depressive symptoms and harsh punishment as potential mediators for the association of IPV with socioemotional development. In the second model, we examined the association between current maternal depressive symptoms and early child socioemotional development and included adjustment for IPV. In the third model, we examined the association between maternal report of current harsh disciplinary practices and child stimulation with socioemotional development as the outcome, including adjustment for IPV. In the final model, we adjusted for both maternal depressive symptoms and harsh discipline in addition to IPV. We used Q-Q plots to assess the normality of residuals and all models appeared to meet the homoscedasticity and normality assumptions of linear regression. All hypothesis tests were 2-sided and *P* values <.05 were considered statistically significant. Missing data were included in the multiple models by creating missing value categories. All analyses were conducted using Stata, version 15 (StataCorp LLC).

## Results

**Figure 2.**
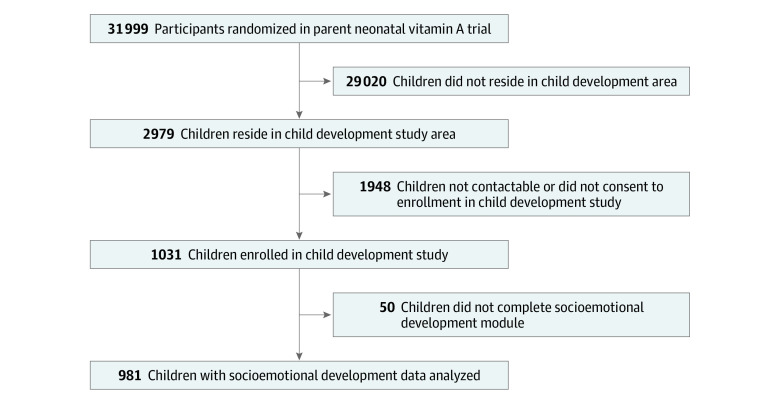
Flow Diagram of Participant Recruitment

A total of 981 mother-child dyads were included in the analytic sample (Figure 2). Overall, 388 of the children (39.6%) were between ages 18 and 24 months (mean [SD] age, 27.06 [6.08] months), 515 were male children (52.5%), and 466 were female children (47.5%). Household, maternal, and child characteristics are presented in Table 1. In this sample, 257 mothers (26.2%) reported experiencing physical and/or sexual IPV and 83 mothers (8.5%) were experiencing mild to severe depressive symptoms. The majority of mothers (876 [89.3%]) reported yelling at their children, 609 (62.1%) mothers spanked or hit their children, and 930 (94.8%) reported using a moderate or low number of stimulation activities. The mean (SD) child CREDI socioemotional development score was 0.73 (0.12).

**Table 1.** Household, Maternal, and Child Characteristics of the Study Population

Characteristic	No. (%)
**Household**	
Head of the family^a^	
No.	960
Mother	52 (5.4)
Father	757 (78.9)
Grandparents	131 (13.6)
Other	20 (2.1)
Father stays in the home^a^	
No.	960
No	148 (15.4)
Yes	810 (84.4)
Not applicable	2 (0.2)
Source of household water^a^	
No.	960
Pipe water	223 (23.2)
Public tap	266 (27.7)
Open well	402 (41.9)
Closed well	47 (4.9)
Surface water	13 (1.3)
Tube well, borehole, or hand pump	9 (1.0)
Latrine^a^	
No.	960
Flush toilet	238 (24.8)
Pit latrine	714 (74.4)
Other	8 (0.8)
No. of rooms in the house^a^	
No.	960
≤2	280 (29.2)
3	287 (29.9)
≥4	393 (40.9)
No. of people that regularly eat in the household^a^	
No.	977
≤3	243 (24.9)
4-5	408 (41.8)
≥6	326 (33.4)
**Mother**	
Age, y^a^	
No.	958
15-24	415 (43.3)
25-34	439 (45.7)
35-44	104 (10.9)
No. of children^a^	
No.	981
1	264 (26.9)
2	247 (25.2)
3	204 (20.8)
≥4	266 (27.1)
Level of education^a^	
No.	957
None/some primary	50 (5.2)
Completed primary	841 (87.9)
Secondary or advanced	66 (6.9)
Lifetime exposure to IPV	
No.	981
None	724 (73.8)
Physical violence	144 (14.7)
Sexual violence	50 (5.1)
Physical and sexual violence	63 (6.4)
Depressive symptoms	
No.	981
None or minimal symptoms (PHQ-9 <5)	898 (91.5)
Mild-to-severe symptoms (PHQ-9 ≥5)	83 (8.5)
Mother yells at the child	
No.	981
No	105 (10.7)
Yes	876 (89.3)
Mother spanks/hits the child	
No.	981
No	372 (37.9)
Yes	609 (62.1)
Child stimulation	
No.	981
High	51 (5.2)
Moderate	529 (53.9)
Low	401 (40.9)
**Child**	
No.	981
CREDI socioemotional development score, mean (SD)	0.73 (0.12)
Age, mo^a^	
No.	980
18-23	388 (39.6)
24-30	249 (25.4)
30-36	343 (35.0)
Sex	
No.	981
Male	515 (52.5)
Female	466 (47.5)

Abbreviations: CREDI, Caregiver-Reported Early Childhood Development Instruments; IPV, intimate partner violence; PHQ-9, Patient Health Questionnaire.

^a^ Missing information.

In the unadjusted analysis, experience of physical IPV only, both physical and sexual IPV, and increased maternal depressive symptoms were significantly associated with lower socioemotional development scores for children (Table 2). Multivariable models are presented in Table 3. In multivariable model 1, physical IPV alone was significantly negatively associated CREDI child socioemotional development scores (mean difference, −0.022; 95% CI, −0.045 to −0.006), and both physical and sexual IPV was also significantly negatively associated CREDI child socioemotional development scores (mean difference −0.045; 95% CI, −0.077 to −0.013). Subsequent models were exploratory and examined the potential factors that mediated the association between IPV and child development outcomes. In model 2, exposure to maternal depressive symptoms was negatively associated with child socioemotional development after adjustment for IPV (mean difference, −0.071; 95% CI, −0.102 to −0.041). In model 3, no associations were observed with exposure to harsh punishment after adjustment for IPV and maternal depressive symptoms. In the final model, maternal depressive symptoms, but not IPV exposure, was negatively associated with early child socioemotional development (mean difference, −0.073; 95% CI, −0.103 to −0.043).

**Table 2.** Unadjusted Association of IPV, Maternal Depressive Symptoms, Harsh Child Discipline, and Child Stimulation With Child CREDI Socioemotional Development Scores

Variable	Mean difference in CREDI score (95% CI)	*P* value
IPV		
None	[Reference]	NA
Physical	−0.022 (−0.044 to −0.000)	.04
Sexual	−0.011 (−0.046 to 0.023)	.53
Both physical and sexual	−0.050 (−0.081 to −0.019)	.002
Maternal depressive symptoms		
Minimal or none	[Reference]	NA
Mild to high	−0.079 (−0.106 to −0.052)	<.001
Mother yells at the child		
No	[Reference]	NA
Yes	0.020 (−0.004 to 0.045)	.10
Mother spanks/hits the child		
No	[Reference]	NA
Yes	0.015 (−0.000 to 0.030)	.06
Child stimulation		
Low	[Reference]	NA
Moderate	0.006 (−0.009 to 0.022)	.40
High	0.011 (−0.024 to 0.046)	.54

Abbreviations: CREDI, Caregiver-Reported Early Childhood Development Instruments; IPV, intimate partner violence; NA, not applicable.

**Table 3.** Multivariable Analysis of Exposure to IPV, Maternal Depressive Symptoms, Harsh Child Discipline, and Child Stimulation and Child CREDI Socioemotional Development Scores

Variable	Mean difference (CI)		
	Model 1: IPV^a^	Model 2: Maternal depression^a^	Model 3: Harsh punishment and stimulation^a^
IPV			
None	[Reference]	[Reference]	[Reference]
Physical	−0.022 (−0.045 to −0.006)^b^	−0.015 (−0.037 to 0.006)	−0.014 (−0.036 to 0.008)
Sexual	−0.012 (−0.048 to 0.023)	−0.002 (−0.037 to 0.032)	−0.000 (−0.036 to 0.034)
Both physical and sexual	−0.045 (−0.077 to −0.013)^c^	−0.025 (−0.058 to 0.007)	−0.021 (−0.054 to 0.012)
Maternal depressive symptoms			
Minimal or none	NA	[Reference]	[Reference]
Mild to high	NA	−0.071 (−0.102 to −0.041)^c^	−0.073 (−0.103 to −0.043)^c^
Mother yells at child			
No	NA	NA	[Reference]
Yes	NA	NA	0.012 (−0.014 to 0.039)
Mother spanks/hits child			
No	NA	NA	[Reference]
Yes	NA	NA	0.12 (−0.004 to 0.030)
Child stimulation			
High	NA	NA	[Reference]
Moderate	NA	NA	0.007 (−0.008 to 0.023)
Low	NA	NA	0.007 (−0.028 to 0.043)
No.	981	981	981

Abbreviations: CREDI, Caregiver-Reported Early Childhood Development Instruments; IPV, intimate partner violence; NA, not applicable.

^a^ Adjusted for child sex, maternal age, maternal educational level, father staying at home, wealth index, and randomized vitamin A or placebo regimen.

^b^ *P* < .05.

^c^ *P* < .001.

## Discussion

This study found that 1 in 4 mothers reported experiencing physical and/or sexual IPV and approximately 1 in 12 reported mild to severe depressive symptoms in rural Tanzania. There was a high prevalence of harsh disciplinary practices of yelling and spanking or hitting, and most mothers reported performing a moderate to low number of stimulation activities. Maternal reports of physical only and physical and sexual IPV were associated with lower child socioemotional scores and was no longer significant after including depressive symptoms in the model, which is consistent with mediation. Furthermore, maternal mild to severe depressive symptoms were associated with lower child socioemotional development scores, including adjustment for IPV. Use of harsh disciplinary practices or level of stimulation were not associated with child socioemotional development after adjusting for IPV, maternal depressive symptoms, and other factors.

We found that maternal report of physical only and physical and sexual IPV associated with lower child socioemotional scores is consistent with the literature. Children may not directly experience the violence, but they may still be affected by the abuse through the state of maternal mental health.^30^ A study in the US observed that children whose mothers were exposed to IPV had delay in at least 1 developmental milestone, increasing almost twice the chance of delay in personal-social development.^15^ Children exposed to IPV are also at increased risk for adverse mental and behavioral health sequelae, as well as effects on the brain and stress regulation systems.^31^ The stress regulation systems and children’s own mental health could be additional pathways for explaining the association between IPV and children’s socioemotional development that were not explored in this study.

We also found that there was no association of maternal report of IPV with socioemotional development after adjustment for depressive symptoms. As a result, this suggests that maternal depression may mediate the association between IPV and socioemotional development that is consistent with our model presented in Figure 1. Beyond physical manifestations, IPV also affects the mental health of those who experience it. A study in Kenya found that caregivers reporting psychological and physical violence were more likely to be at risk for depression.^32^ A systematic review noted that children exposed to IPV had a high prevalence of depressive symptoms, insecurity, and posttraumatic stress disorder, followed by adjustment/behavior problems and aggression, and declining academic performance.^33^ Considering the potential mediation observed in this study, unexplored mother interpersonal and/or contextual factors within maternal depression and IPV should be explored in determining child socioemotional development.

We also found that maternal mild to severe depressive symptoms were associated with lower child socioemotional development scores adjusted for maternal IPV. These findings are consistent with a study conducted in Pakistan reporting that maternal depressive symptoms during the first years of the child’s life were associated with lower child socioemotional development scores.^13^ A systematic review examined the association between maternal depression among Latina mothers and found that children whose mothers were experiencing depression had significantly poorer sociodevelopmental outcomes than children whose mothers were not depressed.^34^ Also, a study with children aged 11 years conducted in Brazil found peer relationship problems and lower levels of prosocial behavior when mothers had persistent depressive symptoms, compared with children whose mothers had low levels of depressive symptoms.^35^ Maternal depressive symptoms can reduce a mother’s emotional readiness to interact with and respond appropriately to their child’s development.^36^ Also, maternal depression may interfere with mothers’ abilities to be nurturing and has a negative influence on parenting practices.^37^ Overall, our findings add to the literature that depressive symptoms are associated with low child socioemotional development scores.

In the study context of rural Tanzania, the majority of mothers had low to moderate engagement of play and learning activities with their young child; however, we did not find an association between stimulation and socioemotional development. Prior research shows a generally positive association between higher levels of early stimulation and improved child development.^38,39,40,41^ A longitudinal study in the UK, with 30 years of follow-up, found positive associations between child stimulation, mental health, and individual resilience.^42^ In particular, socioemotional development is related to the interaction between humans, which may have a devastating impact due to lack of stimulation and when mothers are unwilling to provide warm interactions, especially during the depressive periods.^43^

Finally, an association between harsh discipline and socioemotional development was not found, although most children in the sample received harsh discipline. Prior research has found that when maternal depressive symptoms and IPV coexist, there is an increasing chance of harsh child discipline.^44^ In Nicaragua, a study reported that maternal experience of IPV was associated with a 17% increase in the risk of harsh child discipline.^45^ As a result, even though we did not identify an association between harsh discipline and child socioemotional development in this analysis, after adjusting for IPV and maternal depressive symptoms, there is a growing amount of literature on how harsh discipline can affect child development.^46,47^

### Limitations

This study has limitations. First, this study is from the Morogoro region in Tanzania, which may not be generalizable to the broader Tanzanian context or other settings. Second, the PHQ-9 measures depressive symptoms and is not a diagnostic measure for maternal depression. Third, we did not measure psychological and other forms of IPV (eg, verbal, financial, and legal), which probably would change the prevalence of IPV experienced. Fourth, the indicator for child stimulation measured the quantity of stimulating activities the caregiver engaged in with their child but does not capture the quality of that engagement. Also, no information was collected regarding father (or other caregiver stimulation) for which emerging evidence suggests implications for child socioemotional development.^6^ Fifth, we used only 2 questions to measure harsh discipline. Sixth, we did not account for multiple comparisons for secondary analyses and, as a result, there is a risk of type I errors or falsely finding a significant difference.

## Conclusions

The high burden of IPV and depression indicates the need for interventions for mothers, children, and families in rural Tanzania and similar settings to reduce and ameliorate IPV and poor maternal mental health. In addition, growing up in an abusive and toxic environment can greatly affect the psychosocial development of children and subsequently affect their life outcomes. Improving access to screening for IPV, maternal depressive symptoms, and protective childcare may be warranted. Clear protocols to help health clinicians, social workers, and protective sectors (eg, police officers) in how to identify all forms of IPV and make appropriate referrals to protect the caregiver and child are warranted. Globally, protection policies for mothers and children are needed to address the prevention of violence and to provide safety and support for those who have experienced violence.

## Notes

Supplement.
Data Sharing Statement
