# Supplementary material for: Association of Exposure to Intimate Partner Violence With Maternal Depressive Symptoms and Early Childhood Socioemotional Development Among Mothers and Children in Rural Tanzania
Source: JAMA Netw Open. 2022 Dec 29;5(12):e2248836. doi: 10.1001/jamanetworkopen.2022.48836 (PMC9857043; doi:10.1001/jamanetworkopen.2022.48836)
Supplement: Supplement. — Data Sharing Statement [file jamanetwopen-e2248836-s001.pdf]

Data Sharing Statement

Ramos de Oliveira. Association of Exposure to Intimate Partner Violence With Maternal Depressive Symptoms and Early Childhood Socioemotional Development Among Mothers and Children in Rural Tanzania. *JAMA Netw Open*. Published December 29, 2022.
doi:10.1001/jamanetworkopen.2022.48836

Data
Data available: Yes
Data types: Other (please specify)
Additional Information: The data will be shared if requested.
How to access data: crsudfeld@gmail.com
When available: With publication

Supporting Documents
Document types: None

Additional Information
Who can access the data: researchers whose proposed use of the data has been approved
Types of analyses: For research
Mechanisms of data availability: with investigator support
